# Hyaluronic acid in tobacco-exposed rats. Inflammatory reaction, and duration of effect^1^

**DOI:** 10.1590/s0102-8650201900202

**Published:** 2019-02-28

**Authors:** Cristina Pires Camargo, Renan Dias Frassei, Daniel Imbassahy de Sá Bittencourt Camara e Silva, Robert Zawadzki Pfann, Luiza de Campos Moreira da Silva, Julio Morais-Besteiro, Rolf Gemperli

**Affiliations:** I MD, Division of Plastic Surgery, Hospital das Clínicas, Laboratory of Microsurgery and Plastic Surgery (LIM-04), Medical School, Universidade de São Paulo (USP), Brazil. Intellectual and scientific content of the study, interpretation of data, statistics analysis, manuscript writing, critical revision.; II Graduate student, Laboratory of Microsurgery and Plastic Surgery (LIM-04), Medical School, USP, Sao Paulo-SP, Brazil. Acquisition, analysis and interpretation of data; technical procedures.; III PhD, Division of Plastic Surgery, Hospital das Clínicas, Laboratory of Microsurgery and Plastic Surgery (LIM-04), Medical School, USP, Sao Paulo-SP, Brazil. Intellectual and scientific content of the study, interpretation of data, critical revision.

**Keywords:** Tobacco, Hyaluronic Acid, Rats

## Abstract

**Purpose:**

To evaluate the hyaluronic acid (HA) inflammatory reaction, fibroblasts, fibrosis and duration of effect in the dorsal region of tobacco-exposed rats.

**Methods:**

Ten Wistar rats were divided into two groups: tobacco-exposed-group (TEG;n=5) and air-control-group (CG;n=5). The TEG animals were tobacco-exposed twice a day, 30-minutes/session, during 60 days. After this period, all animals received 0.1 mL HA subcutaneous injection in the dorsal area. The volume of HA was measured immediately after HA injection and weekly using a hand-caliper in nine weeks. After this period, all the animals were euthanized, and a specimen of was collected to evaluate inflammatory cells, fibroblasts, and fibrosis by HE.

**Results:**

This study showed a higher inflammatory reaction in TEG than CG: inflammatory cell-count (CG: 1.07±0.9; TEG: 8.61±0.36, p<0.001); fibroblast count (CG: 2.92±0.17; TEG: 19.14±0.62, p<0.001), and fibrosis quantification (CG: 2.0; TEG: 3.75, p<0.001). The analysis of the HA volume in nine weeks in the dorsal region did not show a difference between groups (p=0.39).

**Conclusions:**

This study suggested that the HA injection in the TEG caused an increase in inflammatory cell count, fibroblast, and fibrosis quantification when compared to the CG. There was no difference in the duration of effect of HA between the groups.

## Introduction

 All implantable substances promote inflammatory reaction represented by an elevation of the inflammatory cell count (acute phase) and fibroblast deposition (long-term phase)^1^.

 The HA is one of the most frequent no-surgical treatment for rejuvenation, due to the low immunogenicity, biocompatibility, and temporary effect (absorbable substance)^2^ ^,^ ^3^.

 Several papers showed minimal inflammatory response in the adjacent subcutaneous tissue^3^ ^-^ ^6^.

 However, some intrinsic and extrinsic factors can increase inflammatory status and ultimately enhance inflammatory response to an implant. For instance, smoke habit causes an inflammatory state in all organs. In the skin, cigarette smoke causes endothelial dysfunction, an increase the synthesis of catecholamines and cause an imbalance of thromboxane and prostaglandins levels. All of these changes cause vasoconstriction, increase of free radicals in the organism^7^ ^-^ ^9^.

 However, until now, we do not have any data regarding the HA inflammatory reaction in smoke subjects (fibrosis, inflammatory reaction).

 For this reason, this study aimed to compare the histological effect of HA in the dorsal region of tobacco-exposed rats and control rats.

## Methods

 This study was approved by the Ethical Committee of the School of Medicine, Universidade de São Paulo (050/16). All animal management was in accordance with the International Council for Laboratory Animal Science.

 We studied ten male Wistar rats, weighing from 200 to 250g. The animals were kept in a vivarium in a 12-h day/night cycle and fed standard feed and water *ad libidum.* The rats were divided into two groups: animal exposed to tobacco smoking (n=5) and air control group (n=5).

 The primary endpoint was the inflammatory reaction of HA effect in the tobacco-exposed animals, inflammatory cell count, fibroblast cell count, and fibrosis quantification in the surrounding injection tissue at the ninth week. 

 The secondary endpoint were: duration of effect measured by a hand caliper immediately after the HA injection every week for nine weeks, HA absorption by time, the difference of HA absorption between groups, and the correlation between tobacco smoking.

###  Tobacco exposure 

 The animals were exposed to smoke in a 28-L plastic box with three orifices: on the inlet for synthetic air (2 L/min); another for smoke; and an outlet to ventilate excess smoke. The smoke inlet was connected to a Venturi System controlled using fluxometry (2.5 L/min), which in turn was connected to a lit cigarette.

 Carbon monoxide (CO) was monitored using a single-gas detector (ToxiPro; Biosystems, USA) to maintain a CO concentration of 300-350 parts per million (ppm) inside the box^10^. The smoke exposure regimen consisted of two daily sessions (30 min per session) for 60 days^11^.

###  Injection procedure 

 All the animals were anesthetized (ketamine 100 mg/kg associated with xylazine 10 mg/kg). We trichotomized a 2×2 cm area along the middle line of the dorsum in the level of the scapula. Antisepsis was performed using chlorhexidine. And we injected subcutaneously 0.1 mL hyaluronic acid (Volift^®^, Allergan, Irvine) in the dorsal region, between the scapula^12^.

###  Microscopic analysis 

 At the end of the ninth week, all animals were euthanized using anesthetic over dosage.

 A 1×1 cm sample through the HA central axis was collected. The sample was fixed in formalin 4% for 24 h. Tissue was washed, dehydrated in graded concentrations of alcohol and embedded in paraffin. Four-micrometer-thick sections were mounted on glass slides and stained with hematoxylin and eosin. The sections were analyzed under the light microscope (Nikon eclipse E600^®^) for description the tissue surrounding the HA.

 The inflammatory response was analyzed by inflammatory cell count (polymorphonuclear and monocyte cells) in 10 fields (x40 magnification) and capsule morphologic description.

 We also counted the fibroblasts in 10 fields (x40 magnification). The fibrosis was measured according to the Likert scale (0=no fibrosis, 1= mild fibrosis, 2=moderate fibrosis, 3=high quantity of fibrosis, 4=severe fibrosis). 

###  Macroscopic analysis 

 We analysed the HA volume during nine weeks, the difference between tobacco-exposed group and control group during nine weeks. 

 According to Hillel *et al.* ^12^, we measured by hand-caliper instrument the three diameters (vertical diameter, horizontal diameter, and high) of the HA bolus injection, immediately HA injection and every week during nine weeks. 

 The diameter was uploaded to Excel software (Windows 7^®^, 2007), and we calculated the volume (mm^3^) of the bolus injection. We plotted the HA bolus volume versus time during nine weeks to compare: the HA absorption by time, the difference of HA absorption between groups.

###  Statistical analysis 

 The sample size was based on Hillel *et al*.^12^ article. We adopted the same sample size (five animals per arm), considering a higher inflammatory response in smoke-exposed group when compared to the control group.

 All variables were tested using descriptive and inferential statistical analyses. The intergroup analysis was done using Wilcoxon rank sum test. The correlation between these two groups was analyzed using the Spearman correlation test, and then we correlated these two variables using the determination coefficient calculation (R^2^). The analysis was done using Stata 14 (StataCorp 2015, Stata Statistical Software: Release 14. College Station, TX, StataCorp LP).

## Results

 All animals survived during the study without any local or systemic complication.

###  Microscopic analysis 

**Figure 1:**
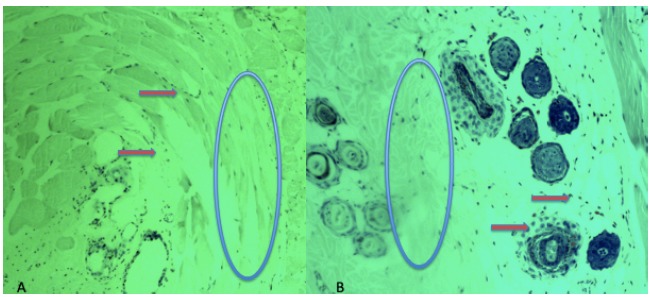
Microscopic analysis. The red arrows showed inflammatory cells around the HA injection. The blue circle showed HA in the subdermal layer (x200, HE). **A**. Control group; **B.** Tobacco-exposed group.

 Microscopic analysis showed a higher inflammatory reaction in the tobacco-exposed group (Table 1, Fig. 1).

**Table 1:** Microscopic analysis and comparison between control and tobacco-exposed groups.

Variable	Control group	Tobacco-exposed group	P-value
Inflammatory cell count (mean±SD)	1.07±0.95	8.61±0.36	<0.001
Fibroblast count (mean±SD)	2.92±0.17	19.14±0.62	<0.001
Fibrosis (median and IQR)	2 (2-2)	3.75 (3.5-4)	<0.001

SD, standard deviation; IQR, interquartile range.

###  Macroscopic analysis 

 The volume of HA measured by hand-caliper showed a decrease in the HA volume in nine weeks in the tobacco-exposed group (p<0.001) and control group (p<0.001).

 However, the HA volume hand-caliper measurement did not show any difference between the tobacco-exposed group and control group was not significant (p=0.39) in nine weeks. 

 Moreover, to certify this measurement we showed a strong coefficient of determination (R^2^) between HA volume and filler degradation in both groups (R^2^=0.88 and R^2^=0.84 in the control and tobacco-exposed groups, respectively).

**Figure 2:**
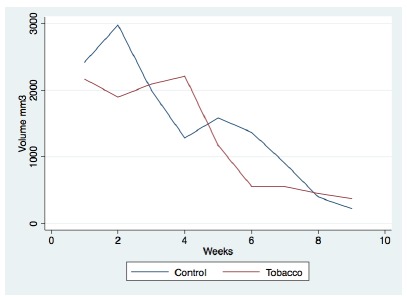
Duration of HA effect (volume in nine weeks).

**Figure 3:**
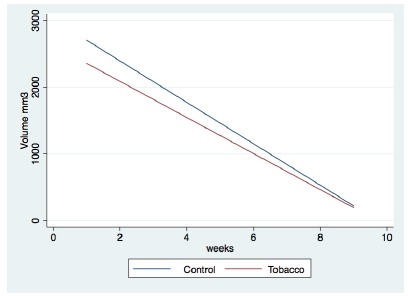
Linear regression: control group, p=0.002, R^2^=0.88; tobacco-exposed group, p=0.006, R^2^=0.84.

 In the control group, a peak volume was shown in the second week, and in the tobacco-exposed group, the peak volume was shown in the fourth week 4 (Figs. 2 and 3).

 This linear regression graphic enabled the following formulas to predict the absorption of HA for each group according to the time unit of the treatment.

Control group:

 Volume= 3013.3 - 310.4*weeks

Tobacco-exposed group:

 Volume=2627.6 - 270.5*weeks

## Discussion

 This study showed a higher inflammatory reaction in TEG than in the Control group. All implantable substances promote inflammatory reaction represented by a high inflammatory cell count (acute phase) and fibroblast deposition (long-term phase)^12^ ^-^ ^14^.

 Regarding HA injection, several papers showed a minimal inflammatory response in the adjacent subcutaneous tissue. These papers showed a few amounts of inflammatory cells and only a few amounts of fibroblasts in the adjacent subdermal tissue^13^ ^,^ ^14^.

 In our study, the control group showed similar results to the literature data, a minimal amount of inflammatory cell count, and minimal fibrosis^13^. 

 Regarding the tobacco-exposed group, the histological analysis showed a higher inflammatory cell count, fibroblast cell count and fibrosis deposition in tobacco-exposed animals group when compared to the control group (p<0.001). 

 Although, there is no literature data about the hyaluronic acid inflammatory reaction in subdermal injection, we hypothesized the tobacco components cause a microenvironment inflammation. In fact, according to literature, smoking habit cause an increase in inflammatory biomarkers in different organ tissues^15,16.^

^^This subclinical inflammatory condition could amplify the immunological and inflammatory reaction by extraneous substances. Therefore, despite the low immunogenicity property, the HA, when injected in a smoker subject, caused a more significant inflammatory response than the control group. Clinically, this fact could explain some palpable nodules after facial filler.

 The secondary outcomes were related to HA absorption. The analysis of the HA volume during nine week showed constant decrease of the HA during this period. Hillel *et al*.^12^ compared the correlation of hand-caliper measurement outcome to the magnetic resonance. These authors showed a strong correlation and concluded the hand-caliper tool was similar to magnetic resonance. For this reason, we adopted hand caliper as a valid tool to measure HA subdermal bolus. 

 We study the HA volume in the subdermal layer for nine weeks. Differently from Hillel *et al.* ^12^, we limited this period based on visual and tactile perception of HA volume in the subdermal layer. After nine weeks the measurement of HA in the control group became difficult. After collecting all the volume data we test the correlation between volume and time. Our study showed a strong correlation (R^2^ greater than 0.8) that reinforce the formula as a way to predict HA absorption time.

 Moreover, we showed in the control group an increase volume (volumetric peak at two weeks after the injection; this result was similar to literature^12^. The reason of this volume increase was the hydrophilic properties of the HA. However, in the tobacco-exposed group, this peak volume was observed in the fourth week. This late outcome in the tobacco-exposed group could be related to a low vascularized environment, due to tobacco effects in the microvasculature structure, and consequently a less water viability in the extracellular matrix^12^ ^,^ ^14^.

 Another point to discuss was the smoke-exposure model and period. We adopted this model to mimic a moderate smoker patient. According to our previous study this model induced a carboxyhemoglobin level similar to a moderate smoker^17^ ^,^ ^18^. Moreover, we exposed the animal for two-months to cause a tobacco endothelial dysfunction^11^ ^,^ ^17^.

 This study had some limitations, one of the drawbacks of the rat model was the difference in the anatomical structure between human subcutaneous tissue and panniculus carnosus at the rat^11^. Considering these differences and according to Marler *et al.* ^19^, the best alternative to mimic human dermal injection was intradermal HA injection in rats.

## Conclusions

 This study suggested that the HA injection in the tobacco-exposed group showed an increase in inflammatory cell count, fibroblast count, and fibrosis quantification when compared to the control group. There was no difference in the duration of effect of injected HA in the tobacco-exposed and control group.
